# Necrotizing Fasciitis With Pelvic Osteomyelitis: A Fulminant Disease

**DOI:** 10.7759/cureus.96772

**Published:** 2025-11-13

**Authors:** Inês Silveira, Raquel Azevedo, Soraia G Araújo, Martinha M Vale, Ana Oliveira

**Affiliations:** 1 Intensive Care Unit, ULS Braga, Braga, PRT; 2 Infectious Disease, ULS Braga, Braga, PRT; 3 Critical Care Medicine, ULS Braga, Braga, PRT; 4 Internal Medicine, ULS Braga, Braga, PRT

**Keywords:** diabetes mellitus, fungal coinfection, necrotizing fasciitis, pelvic osteomyelitis, polymicrobial infection

## Abstract

Necrotizing fasciitis (NF) is a rare, rapidly progressive soft-tissue infection associated with high morbidity and mortality. Diabetes, obesity, and immunosuppression are well-known risk factors that predispose to severe disease and atypical presentations. Complications such as pelvic osteomyelitis and fungal coinfection are exceptionally uncommon in NF and are associated with markedly worse outcomes. Early recognition and immediate surgical intervention are critical to improving survival.

We report the case of a 64-year-old woman with poorly controlled type 2 diabetes, obesity, and baseline gait limitation due to degenerative disc disease, who presented with severe prostration and inability to walk for one week. On admission, she was hypotensive, tachycardic, and confused. The right lower limb was poorly perfused, externally rotated, and exhibited crepitus.

Laboratory studies revealed leukocytosis, elevated C-reactive protein, and acute kidney injury. Computed tomography (CT) demonstrated extensive subcutaneous and intermuscular emphysema of the anterior abdominopelvic wall with caudal extension to both legs, along with pelvic bone changes consistent with osteomyelitis. A diagnosis of septic shock secondary to NF with pelvic osteomyelitis was established. Empiric broad-spectrum antibiotics with piperacillin-tazobactam, clindamycin, and vancomycin were initiated immediately, followed by emergency surgery on the day of admission and subsequent procedures, including amputation and right hip disarticulation. Intraoperative cultures revealed a polymicrobial infection involving bacterial and fungal organisms, including *Candida tropicalis. *Antibiotic therapy was subsequently adjusted, and fluconazole was added for antifungal coverage. Despite repeated debridement, broad-spectrum antibiotics, antifungal therapy, and vasopressor support, the infection progressed, resulting in the patient’s death.

NF can progress insidiously in diabetic patients, particularly those with neuropathy, and may present atypically with diminished pain and less pronounced tenderness, delaying diagnosis. Osteomyelitis is a rare but severe complication reflecting contiguous extension. Fungal involvement, though uncommon, may occur and is associated with increased severity and mortality.

Prompt recognition, early empiric broad-spectrum antibiotic therapy, and immediate surgical source control, which remains the cornerstone of management, are essential. This case highlights the devastating consequences of delayed recognition of NF in high-risk patients and illustrates its rare association with pelvic osteomyelitis and fungal coinfection.

## Introduction

Necrotizing fasciitis (NF) is a rare, life-threatening infection characterized by rapidly progressive necrosis of fascia and subcutaneous tissues, with reported mortality rates ranging from 20% to over 40%, depending on comorbidities, microbiology, and delays in diagnosis and treatment [1]. Diabetes mellitus, obesity, and immunosuppression are well-established risk factors and are associated with increased risk of severe disease and worse outcomes. Early symptoms are often nonspecific, and diagnosis is frequently delayed, especially in patients with diabetic neuropathy, which can mask pain and other classic features [2]. Rapid initiation of empiric broad-spectrum antibiotics and urgent surgical debridement are essential for survival and are the mainstays of management [3]. We present a fulminant case of NF complicated by pelvic osteomyelitis and polymicrobial infection in a diabetic patient, underscoring the challenges of early recognition and management.

## Case presentation

A 64-year-old woman presented to the Emergency Department with severe prostration and inability to walk for one week. Past medical history included poorly controlled type 2 diabetes with retinopathy, nephropathy, and neuropathy, grade 1 obesity, and degenerative disc disease, which had previously caused significant gait difficulty. As a result, the patient’s family delayed seeking medical care when her condition worsened.

On arrival, approximately seven days after symptom onset, the patient was confused, hypotensive (72/41 mmHg), and tachycardic (132 bpm). Physical examination revealed the right lower limb to be poorly perfused, externally rotated, and exhibiting crepitus, raising immediate suspicion for a deep soft-tissue infection such as NF. Laboratory evaluation showed leukocytosis with neutrophilia, markedly elevated C-reactive protein, and serum creatinine of 2.5 mg/dL. The calculated Laboratory Risk Indicator for Necrotizing Fasciitis (LRINEC) score was 9, indicating a high likelihood of NF.

**Figure 1:**
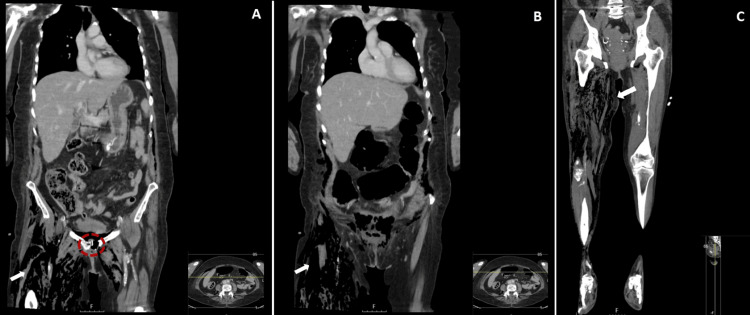
Coronal CT images of the abdomen, pelvis, and lower limbs (A–C) Sequential coronal CT images showing subcutaneous and intermuscular emphysema extending from the anterior abdominopelvic wall to the thighs (white arrows). Cortical discontinuity and osseous pneumatosis of the pubic symphysis are also observed (red dashed circle). These findings are consistent with NF and associated osteomyelitis.

**Figure 2:**
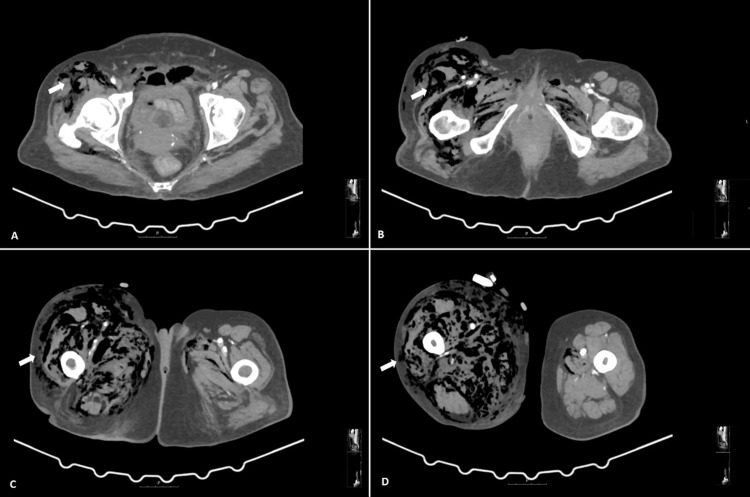
Axial CT images of the pelvis and thighs (A–D) Axial CT slices demonstrating extensive subcutaneous and intermuscular emphysema of the bilateral anterior abdominopelvic wall, with gaseous foci extending to the right iliopsoas muscle and both thighs (white arrows), consistent with NF.

Computed tomography (CT) of the abdomen and pelvis revealed extensive subcutaneous and intermuscular emphysema of the anterior abdominopelvic wall extending into both legs, along with heterogeneity of the pubic symphysis, cortical discontinuity, and osseous pneumatosis-findings consistent with NF and associated pelvic osteomyelitis (Figures 1-2).

Broad-spectrum empiric antibiotics (piperacillin-tazobactam, clindamycin, and vancomycin) were initiated immediately upon admission. The patient underwent emergency surgery on the same day, with extensive fascial and muscular debridement of the right lower limb and anterior abdominopelvic wall. Due to rapid clinical deterioration, a second emergent operation was performed the following day, consisting of right lower limb amputation. On the third day after admission, persistent necrosis necessitated a third procedure involving right hip disarticulation and further pelvic debridement. Intraoperative cultures revealed a polymicrobial infection involving *Streptococcus anginosus*, *Streptococcus constellatus*, *Actinomyces funkei*, *Peptostreptococcus species*, *Stenotrophomonas maltophilia*, and *Candida tropicalis*. Based on antimicrobial susceptibility results, antibiotic therapy was subsequently adjusted to piperacillin-tazobactam and clindamycin, with fluconazole added to target the C. tropicalis isolate.

Despite aggressive surgical and medical management, including multiple debridements, broad-spectrum antibiotics, antifungal therapy, and vasopressor support, the infection continued to progress, ultimately resulting in the patient’s death.

## Discussion

NF is a rare but severe soft-tissue infection characterized by rapidly progressive necrosis of fascia and subcutaneous tissue, with high morbidity and mortality rates [1]. Mortality largely depends on patient comorbidities, timeliness of intervention, and microbial etiology. Based on microbiologic features, NF is classified as polymicrobial (type I) or monomicrobial (type II). Polymicrobial infections are more frequent and commonly associated with elderly patients or those with underlying conditions such as diabetes [2].

Our case is notable for two uncommon aspects. First, the development of pelvic osteomyelitis represents an extremely rare complication of NF, with less than five cases described in the literature [4-7]. Similar to previously published reports, the infection extended deeply into the pubic bone, demonstrating the potential for NF to involve osseous structures, especially in patients with diabetes or immunosuppression [4-6]. Osteomyelitis in this setting typically necessitates radical surgical management, including wide debridement or even amputation, as was required in our patient [4-7].

Second, the isolation of *C. tropicalis* alongside multiple bacterial pathogens represents an unusual but increasingly recognized finding [8]. *C. tropicalis* has emerged as a notable opportunistic pathogen in diabetic and immunocompromised patients, particularly after prolonged antibiotic exposure or multiple surgical procedures, and is associated with higher morbidity and mortality [9]. Fungal involvement in necrotizing soft-tissue infections, while uncommon, has been documented in both case reports and larger series, most often involving *Candida species* (especially *C. tropicalis* and *C. albicans*), with *Aspergillus* and *Mucorales* species also reported [9,10]. In a large surgical series, 10.7% of patients with necrotizing soft-tissue infections had positive intraoperative fungal cultures, predominantly as mixed bacterial-fungal infections; these patients experienced a three-fold increase in mortality and required more surgical interventions compared to those without fungal involvement [10]. In our patient, long-standing diabetes with microvascular complications, repeated surgical debridements, vasopressor use, and critical illness likely predisposed to fungal coinfection.

Diagnostic delay plays a major role in prognosis. Early clinical signs may be subtle, and the absence of fever or typical cutaneous changes often leads to diagnostic uncertainty. In diabetic patients with neuropathy, pain may be minimal or absent, further delaying medical evaluation [2,3]. In our case, these factors, combined with baseline mobility limitations, led to a significant delay in seeking care. On presentation, crepitus was the most specific clinical clue, while CT imaging was crucial in defining the extent of disease and identifying pelvic bone involvement, consistent with previous reports [4-7].

Given these diagnostic challenges, timely recognition and management are crucial. Several laboratory-based tools have been proposed to aid in the early detection of NF. Among them, the LRINEC score integrates C-reactive protein, white blood cell count, hemoglobin, sodium, creatinine, and glucose to stratify risk [11]. In patients with diabetic nephropathy or renal impairment, baseline elevations in creatinine and glucose, along with electrolyte imbalances, can falsely increase the LRINEC score, thereby reducing its specificity for NF [12-14]. In our patient, the calculated LRINEC score was 9, consistent with a high risk of NF. While scores above 8 have been associated with greater disease severity and increased likelihood of amputation, the prognostic value of the LRINEC score remains limited. Recent studies suggest only moderate predictive accuracy for lethality and emphasize that clinical context, hemodynamic status, and comorbidities provide stronger indicators of outcome than laboratory indices alone [15]. Nevertheless, in this case, the markedly elevated LRINEC score was consistent with the fulminant clinical course and fatal outcome, reflecting the severity of systemic illness despite its potential confounders.

When NF is suspected, empiric broad-spectrum antibiotic therapy should be initiated immediately, providing coverage for gram-positive cocci (including methicillin-resistant *Staphylococcus aureus* (MRSA)), gram-negative bacilli, and anaerobes. Piperacillin-tazobactam combined with clindamycin and vancomycin remains a recommended empiric regimen, particularly in diabetic or immunocompromised hosts [2,16]. Clindamycin is especially valuable for its ability to inhibit toxin production by *Streptococcus *and *Staphylococcus species*. Once microbiologic results are available, therapy should be refined according to pathogen susceptibility and clinical response [2,16]. In our case, antimicrobial adjustment to piperacillin-tazobactam and clindamycin, along with the addition of fluconazole to address *C. tropicalis*, reflected this practice.

Early and aggressive surgical debridement remains the cornerstone of treatment and is strongly associated with improved survival, as delays in surgery are the most significant modifiable risk factor for mortality [1-3]. International consensus guidelines from the World Society of Emergency Surgery, the Surgical Infection Society Europe, and the American Association for the Surgery of Trauma emphasize that surgical intervention should occur as soon as NF is suspected, ideally within 6 hours of hospital admission, with repeated debridements as necessary [17]. Our patient underwent multiple extensive procedures, including fasciectomy, amputation, and right hip disarticulation, highlighting the complexity and aggressiveness required to control the infection.

In retrospect, the patient’s presentation with systemic deterioration, poor limb perfusion, and crepitus, despite minimal pain, reflected an already advanced stage of NF. The delay in seeking medical care, influenced by her preexisting mobility limitations and diabetic neuropathy, contributed significantly to the late presentation and poor outcome. In similar high-risk patients, unexplained systemic deterioration, or localized findings such as skin discoloration or crepitus, even in the absence of pain or fever, should prompt early imaging and surgical evaluation.

This case highlights the diagnostic and therapeutic challenges of NF in diabetic patients with neuropathy. The coexistence of pelvic osteomyelitis and fungal coinfection reflects a deep, aggressive infection associated with poor prognosis, underscoring the importance of early clinical suspicion, prompt imaging, timely empiric broad-spectrum antibiotic therapy, and immediate surgical intervention to optimize outcomes.

## Conclusions

This case illustrates the fulminant nature of NF complicated by pelvic osteomyelitis and fungal coinfection in a diabetic patient with delayed diagnosis. Despite aggressive surgical and medical management, the infection remained refractory and resulted in the patient’s death. Early recognition and prompt multidisciplinary intervention are essential to reduce mortality in high-risk patients.
